# International benchmarking of secondary fracture prevention in older adults with hip fracture: a Chinese hospital-based KPI audit

**DOI:** 10.3389/frhs.2026.1799272

**Published:** 2026-05-14

**Authors:** Yuan Yuan, Wei Wang, Ping-Yang Li, Meng Zhang, Yan-Na Lu, Ming-Hui Yang, Wei Tian, Jing Zhang

**Affiliations:** 1 Department of Geriatrics, Beijing Jishuitan Hospital, Capital Medical University, Beijing, China; 2 National Center for Orthopaedics, Beijing, China; 3 Peking University Health Science Center, Beijing, China; 4 Outpatient Department, Beijing Jishuitan Hospital, Capital Medical University, Beijing, China; 5 Department of Traumatic Orthopedics, Beijing Jishuitan Hospital, Capital Medical University, Beijing, China; 6 School of Public Health, Harbin Medical University, Harbin, Heilongjiang, China

**Keywords:** aging population, fracture liaison service, hip fracture, orthogeriatric care, secondary fracture prevention

## Abstract

**Objective:**

This study provides the first international benchmarking of postoperative hip fracture care in Chinese tertiary hospitals using the UK Fracture Liaison Service (FLS) Key Performance Indicators within a “cascade of care” framework.

**Methods:**

This study was reported in accordance with the Strengthening the Reporting of Observational Studies in Epidemiology (STROBE) guidelines. We conducted a retrospective audit of 2,689 patients aged ≥60 years who received hip fracture surgery in an orthogeriatric co-managed ward from 2016 to 2018. Performance was measured against seven validated FLS KPIs: bone mineral density (BMD) screening, anti-osteoporosis medication initiation, falls risk assessment, rehabilitation provision, follow-up adherence, and secondary fracture incidence at specified time points.

**Results:**

Inpatient metrics were excellent: 97.1% (2,610/2,689) underwent BMD screening and 94.0% (2,454/2,610) received bone-protective medication prior to discharge, both exceeding UK benchmarks. Post-discharge follow-up declined sharply: 50.7% (1,363/2,689) at 90 days, 17.7% (477/2,689) at 12–16 weeks, and 9.5% (255/2,689) at 12 months. Secondary fracture rates remained high (11.0–13.4%) across follow-up intervals despite strong treatment initiation. International comparison highlighted strengths in acute inpatient management but exposed deficits in community-based coordination and long-term adherence relative to integrated FLS models.

**Conclusion:**

This pioneering international comparison demonstrates that Chinese hospital-centric orthogeriatric co-management delivers outstanding acute care but struggles with sustained secondary fracture prevention due to fragmented healthcare pathways and limited primary care integration. Implementation of dedicated FLS coordinators, digital engagement tools, and structured hospital–community partnerships is essential to build a comprehensive secondary prevention network and reduce long-term fracture risk in China's aging population.

## Introduction

With China's population aging at an unprecedented pace, fragility hip fractures have become a major public-health concern, imposing complex peri-operative demands, elevated mortality and disability rates, and rising direct costs (1, 2). Preventing secondary fractures is therefore a critical postoperative objective and hinges on timely osteoporosis assessment, targeted anti-resorptive therapy, and structured rehabilitation to restore mobility (3, 4).

Introduced in Europe during the late 1990s, the Fracture Liaison Service (FLS) model systematically identifies individuals at risk for fragility fractures and coordinates multidisciplinary interventions to avert subsequent events (1, 5). Supported by robust evidence, FLS programs have reduced re-fracture risk by 33%–82% over 2–4 years and lowered associated mortality by up to 35% (6, 7). To monitor service quality, England and Wales maintain the web-based Fracture Liaison Service Database (FLS-DB), a national audit embedded within the Falls and Fragility Fracture Audit Programme (FFFAP), which benchmarks care using 11 key performance indicators (KPIs) spanning osteoporosis diagnosis, pharmacologic management, and falls assessment [The Falls and Fragility Fracture Audit Programme (FFFAP)].

**Figure 1:**
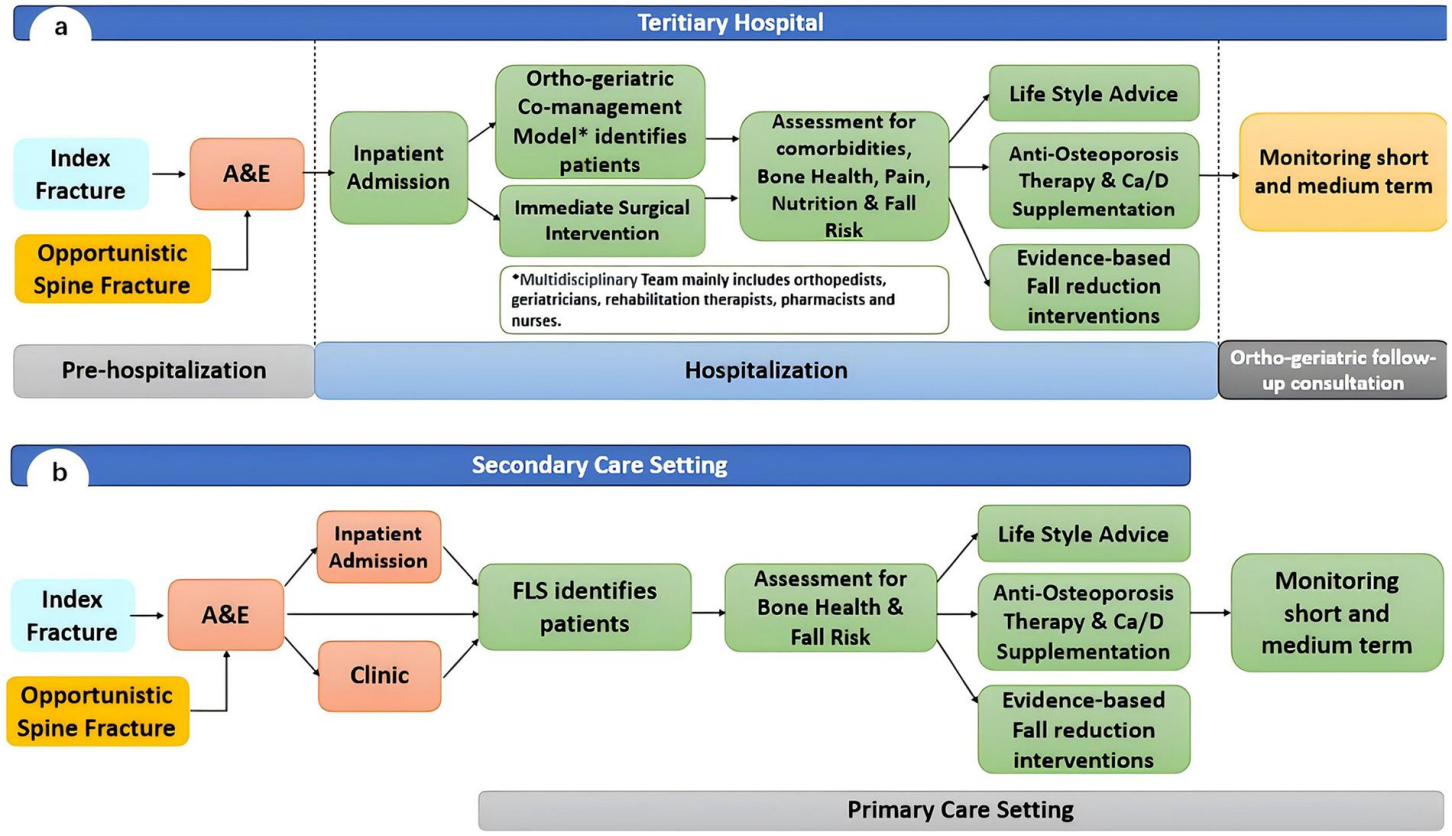
Common FLS flowcharts of China **(a)** and Europe **(b)**.

In our tertiary hospital's pathway for hip fracture care, a team of orthopedic surgeons and geriatricians jointly oversees peri-operative management, inpatient osteoporosis treatment, and rehabilitation initiation (8). Unlike FLS networks in high-income settings, this care pathway remains hospital-centric and seldom integrates primary-care facilities, leaving its impact on secondary-prevention targets uncertain (8, 9) (see Figure 1).

To bridge this knowledge gap, this study conducts the first benchmark audit in China, guided by the FLS-DB KPIs, to assess the performance of hospital-driven secondary fracture prevention against international standards. Rather than evaluating a specific program, this audit aims to characterize the current performance profile—identifying strengths, gaps, and structural particularities. Thereby, it seeks to generate actionable insights for scaling effective secondary-prevention strategies across China.

## Methods

### Ethical approval and reporting guidelines

All patient data were anonymized, and informed consent was waived given the retrospective observational study design, in accordance with institutional review board guidelines. This study was designed and reported following the Strengthening the Reporting of Observational Studies in Epidemiology (STROBE) guideline. The completed STROBE checklist is provided as a Supplementary File.

### Study design

This retrospective audit was conducted through systematic extraction of patient records from the hospital information management system to evaluate the implementation of standardized postoperative management protocols for elderly patients with fragility hip fractures. This study constitutes the initial audit report summarizing data from three consecutive years (2016–2018). Thereafter, annual audits were implemented to examine the ongoing performance.

### Study setting and participants

Patients with acute fragility hip fractures admitted to the orthopedic-geriatric co-managed ward at our hospital from January 2016 to December 2018 were included in this analysis. Clinical data encompassing osteoporosis screening, falls risk assessment, rehabilitation evaluation, anti-osteoporosis treatment protocols, scheduled outpatient follow-up visits, and prescriptions for bone-protective medications were systematically collected from the index fracture admission through 12 months post-fracture.

### Patient selection criteria

The inclusion criteria were (1) patients aged 60 years or older; (2) hip fracture confirmed by radiological imaging (x-ray or computed tomography); (3) injury occurring within 21 days of hospital admission. Exclusion criteria included (1) patients younger than 60 years; (2) pathological fractures secondary to malignancy or high-energy trauma fractures not consistent with fragility fracture mechanisms.

### Data collection protocol

Comprehensive demographic and clinical characteristics were systematically captured, including patient gender, age, fracture classification, surgical procedures, length of hospital stay, medical comorbidities, therapeutic interventions, and clinical outcomes during both the inpatient period and structured follow-up visits. All data were extracted using standardized case report forms to ensure consistency and completeness.

### Orthogeriatric co-management model implementation

To optimize secondary fracture prevention in elderly patients with hip fractures, postoperative management was guided by a structured protocol based on evidence-based best practices. During hospitalization, all patients received individualized rehabilitation programs supervised by a multidisciplinary team comprising physiotherapists, geriatricians, and specialized nursing staff, with the primary objective of facilitating early mobilization and preventing perioperative complications. Concurrent patient education was delivered by geriatricians and geriatric nurses, focusing on osteoporosis management, fall prevention strategies, and the critical importance of long-term adherence to bone-protective therapies.

Anti-osteoporosis medication initiation was recommended prior to discharge. A standardized discharge protocol included scheduled orthopedic-geriatric follow-up appointments, during which radiological assessments, medication adherence reviews, and osteoporosis treatment prescriptions were conducted as clinically indicated. Post-discharge monitoring was primarily conducted through specialized clinic visits co-led by orthopedic surgeons and geriatricians, with geriatricians assuming primary responsibility for overseeing the implementation and monitoring of osteoporosis treatment protocols. Medication adherence was assessed by reviewing outpatient prescription records as well as patient self-report at scheduled follow-up visits.

### Key performance indicators framework

This study adopted 11 internationally validated key performance indicators (KPIs) from the United Kingdom's Fracture Liaison Service Database (FLS-DB) Audit Standards, as outlined in Table 1, to benchmark the quality of our “cascade of care” postoperative management framework. However, four KPIs were excluded from the final analysis due to institutional data limitations. Specifically, KPI 1 (FLS data completeness assessment) was excluded because validated thresholds for defining adequate data completeness within our institutional database had not been established at the time of study initiation.

**Table 1:** The 11 key performance indicators and the calculations.

KPIs	In the FLS-DB (FFFAP)	In our study
**KPI 1**, Data completeness	FLSs with a good level of data completeness	NA
**KPI 2**, Identification of non-spine fractures	The percentage of patient records submitted compared with the local estimated caseload	NA
**KPI 3**, Identification of spine fractures	The percentage of patients with a spine fracture as their index fracture site was calculated and compared with the local estimated caseload	NA
**KPI 4**, Time to FLS assessment	The percentage of patients who were assessed within 90 days of their fracture **Numerator**—people who have had an assessment or the index fracture that led to FLS contact was diagnosed within 90 days **Denominator**—total of all records entered	
**KPI 5**, Time to DXA	The percentage of patients who had a DXA ordered or recommended and were scanned within 90 days of fracture **Numerator**—number of records where difference between the two dates below was less than 90 days:① The date on which the index fracture that led to FLS contact was diagnosed; ② Date of DXA **Denominator**—number of patients who had a DXA ordered or recommended	Due to the widely use of quantitative computed tomography (QCT) in China, we calculated two metrics: ① the original FLS-DB definition restricted to DXA to preserve international comparability; ② the adjusted indicator, defined as the percentage of patients who underwent DXA or QCT scans within 90 days **Numerator**—number of people who were scanned within 90 days **Denominator**—number of patients who had a scan ordered or recommended (scanning defined as DXA only for the original metric, and as DXA or QCT for the adjusted metric)
**KPI 6**, Falls assessment	The percentage of patients who received falls assessment or were referred or recommended for falls assessment **Numerator**—number of people who receive or were referred for a falls risk assessment **Denominator**—total of all records entered	
**KPI 7**, Bone therapy recommended	The percentage of patients who were recommended anti-osteoporosis medication	
	**Numerator**—Number of records where the Question “Bone therapy recommended following index fracture” equals any of the following: ① Referred to GP to decide prescription, ② Referred for further clinical opinion,③ Any named bone-protection treatment **Denominator**—all records submitted	**Numerator**—number of people recommended any bone-protection treatment **Denominator**—all records submitted
**KPI 8**, Strength and balance training	The percentage of non-hip fracture patients over 75 who had started strength and balance training within 16 weeks of their fracture	NA
**KPI 9**, Monitoring contact 12–16 weeks post fracture	The percentage of patients who were followed up within 16 weeks of their fracture	
	**Numerator**—number of patients followed up post fracture = yes **Denominator**—Number of records where question “Bone therapy recommended following index fracture” equals any of the following: ① Referred to GP to decide prescription, ② Referred for further clinical opinion, ③ Any named bone-protection treatment, AND “follow up” is NOT “patient died”	**Numerator**—number of patients followed up within 16 weeks of their fracture **Denominator**—all records submitted
**KPI 10**, Commenced bone therapy by first follow up	The percentage of patients who had commenced (or were continuing) anti-osteoporosis medication within 16 weeks of their fracture **Numerator**—patient records with: Bone protection therapy started = Any named bone-protection treatment and where the difference between the two dates below is between 12 and 20 weeks of ① The date on which the index fracture that led to FLS contact was diagnosed, ② Date of FLS 16 week assessment **Denominator**—number of records where “Bone therapy recommended following index fracture” equals any of the following: ① Referred to GP to decide prescription, ② Referred for further clinical opinion, ③ Any named bone-protection treatment	The percentage of patients who had commenced (or were continuing) anti-osteoporosis medication within 16 weeks of their fracture **Numerator**—patient records with any bone protection therapy started within 16 weeks of their fracture **Denominator**—all records submitted
**KPI 11**, Adherence to prescribed anti-osteoporosis medication at 12 months post fracture	The percentage of patients who had confirmed adherence to a prescribed anti-osteoporosis medication at 12 months post fracture **Numerator**—number of people indicating that they are taking any named bone-protection treatment correctly as prescribed between 48 and 56 weeks follow-up **Denominator**—number of records	

KPI, key performance indicators; FLS-DB, fracture liaison service database; FFFAP, the falls and fragility fracture audit programme; DXA, dual energy x-ray absorptiometry; GP, general practitioner.

KPI 2 (identification of non-spine fractures relative to local estimated caseload) and KPI 3 (identification of spinal fractures relative to local estimated caseload) were not assessed, as accurate and reliable local caseload estimates for fragility fractures were unavailable from hospital administrative datasets or regional health information systems. Additionally, KPI 8 (strength and balance training initiation within 16 weeks for non-hip fracture patients over 75 years) was excluded because our data collection was specifically restricted to hip fracture patients, and systematic documentation of strength and balance training for non-hip fracture patients was not maintained within our electronic health record system. These exclusions were predetermined prior to study commencement to ensure methodological transparency. The seven KPIs assessed in this study were process-based indicators.

### Statistical analysis

Performance assessment against the seven applicable KPIs was conducted using R software for comprehensive data analysis. Descriptive statistics were employed to characterize patient demographics and clinical outcomes, with categorical variables presented as frequencies and percentages, and continuous variables as means with standard deviations or medians with interquartile ranges, as appropriate for data distribution.

## Results

### Patient characteristics and follow-up completion

**Figure 2:**
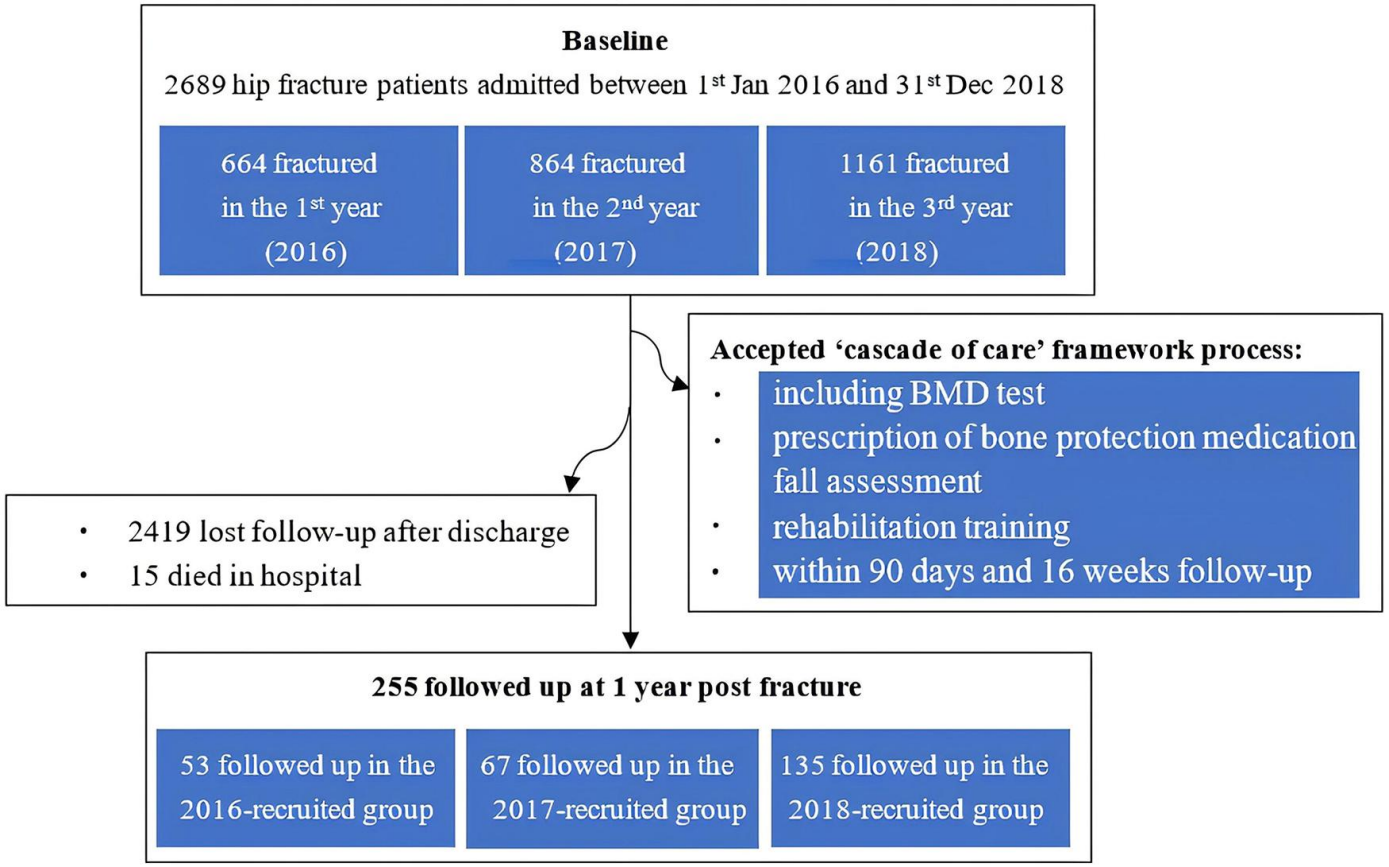
Flowchart of participants recruitment for the audit program.

This audit included 2,689 patients with acute hip fractures who were admitted to the orthogeriatric co-managed ward between January 2016 and December 2018. Fifteen patients died during hospitalization; post-discharge mortality data were unavailable. The attrition rate was substantial, with only 255 patients (9.5%) completing the full 12-month follow-up assessment (see Figure 2).

The median age of the cohort was 80.0 years [interquartile range (IQR) 75.0–85.0]. Female patients comprised 71.7% of the cohort (*n* = 1,929). Femoral neck fractures represented the most common type (54.4%, *n* = 1,438), followed by intertrochanteric fractures (44.6%, *n* = 1,170), mirroring established epidemiological patterns in elderly populations.

The most prevalent comorbidities were hypertension (53.1%, 1,428), diabetes (26.1%, 702), coronary artery disease (18.7%, 503), and cerebrovascular diseases (14.5%, 391) (see Table 2). This comorbidity profile is characteristic of the complex medical conditions typically encountered in elderly hip fracture patients requiring orthogeriatric co-management.

**Table 2:** Characteristics of recruited participants.

Variable	Total *N* = 2,689	2016 *n* = 664	2017 *n* = 864	2018 *n* = 1,161
Age, years, Median (IQR 25,75)	80.0 (75.0, 85.0)	80.0 (75.0, 84.0)	80.0 (75.0, 85.0)	81.0 (75.0, 85.0)
Gender (female)	71.7	73.9	69.9	71.8
Types of hip fracture, %				
Femoral neck fracture	54.4	51.1	53.1	57.2
Intertrochanteric fracture	44.6	47.9	45.6	42.0
Subtrochanteric fractures	1.0	1.0	1.3	0.8
Comorbidities, %				
Hypertension	53.1	55.4	52.3	52.4
Diabetes	26.1	27.3	28.1	23.9
Coronary artery disease	18.7	19.0	18.5	18.7
Cerebrovascular disease	14.5	14.5	17.5	12.4
Dementia/Cognitive impairment	11.2	7.7	12.5	12.1
Cancer/carcinoma	4.5	3.3	3.1	6.1
Parkinson disease	1.6	1.5	1.0	2.0
Chronic respiratory disease	3.3	4.7	2.8	2.8
Anxious or depression	1.2	0.5	1.6	1.4

### Cascade of care performance and secondary fracture outcomes

**Figure 3:**
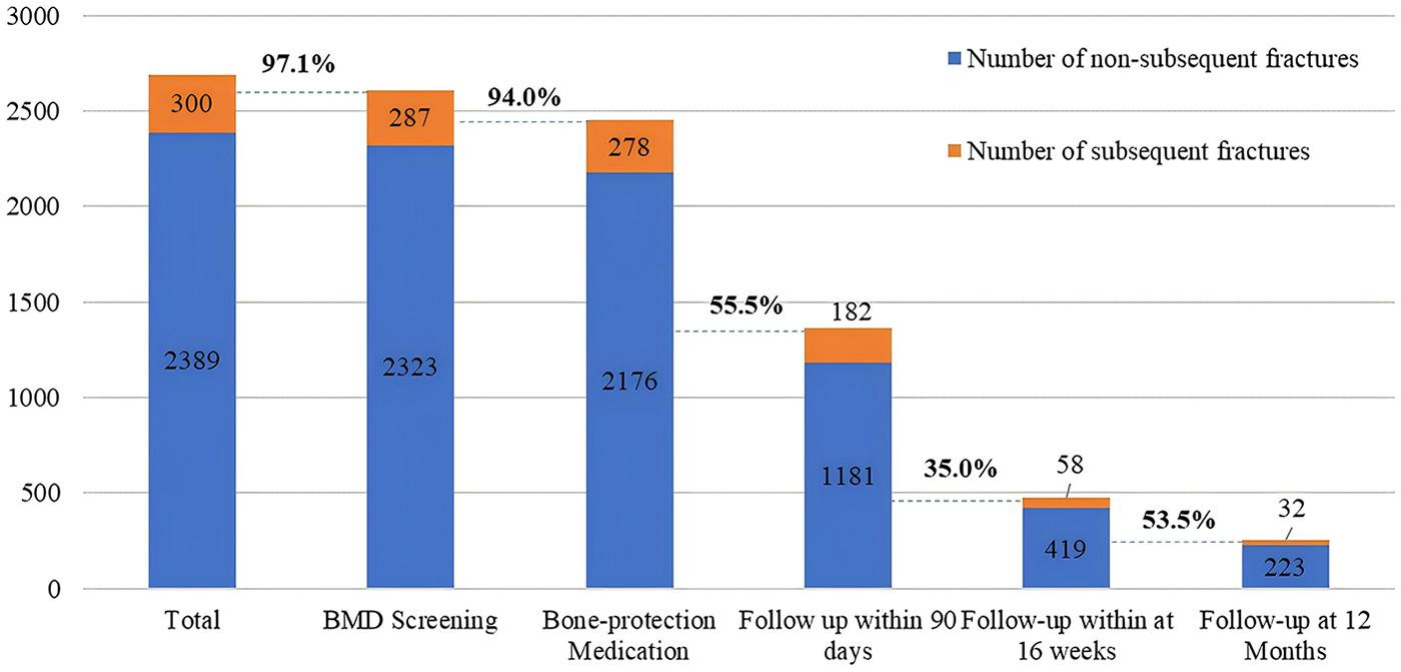
Postoperative management of older patients with hip fracture adopting “cascade of care” framework (cumulative follow-up).

Figure 3 demonstrates the performance of our hospital's postoperative management using the “cascade of care” framework for secondary fracture prevention. Among the 2,689 patients, 2,610 (97.1%) underwent bone mineral density (BMD) screening during hospitalization. Of those screened, 287 (11.0%) experienced a subsequent fracture during the follow-up period, and 2,454 (94.0%) received bone-protective medication prescriptions before discharge. Among patients who initiated therapy, the median duration of calcium/vitamin D supplementation was 45 days (IQR, 4–143), and the median duration of calcitonin therapy was 57 days (IQR, 41–320). The subsequent fracture rate among treated patients was 11.3% (*n* = 278/2,454), indicating persistent fracture risk despite therapeutic intervention.

Progressive attrition was observed throughout the follow-up cascade. At the first follow-up assessment (within 90 days), 1,363 patients attended appointments, with 13.4% (*n* = 182/1,363) sustaining new fractures. By the second follow-up (12–16 weeks post-fracture), attendance declined to 477 patients, with a secondary fracture rate of 12.2% (*n* = 58/477). Finally, 255 patients completed the third follow-up (16 weeks to 12 months), with 12.5% (*n* = 32/255) experiencing subsequent fractures.

These findings reveal a consistent pattern of declining follow-up adherence over time, while subsequent fracture rates remained persistently high across all assessment stages, highlighting the ongoing challenge of maintaining patient engagement in secondary prevention programs.

### Benchmarking against international key performance indicators

**Figure 4:**
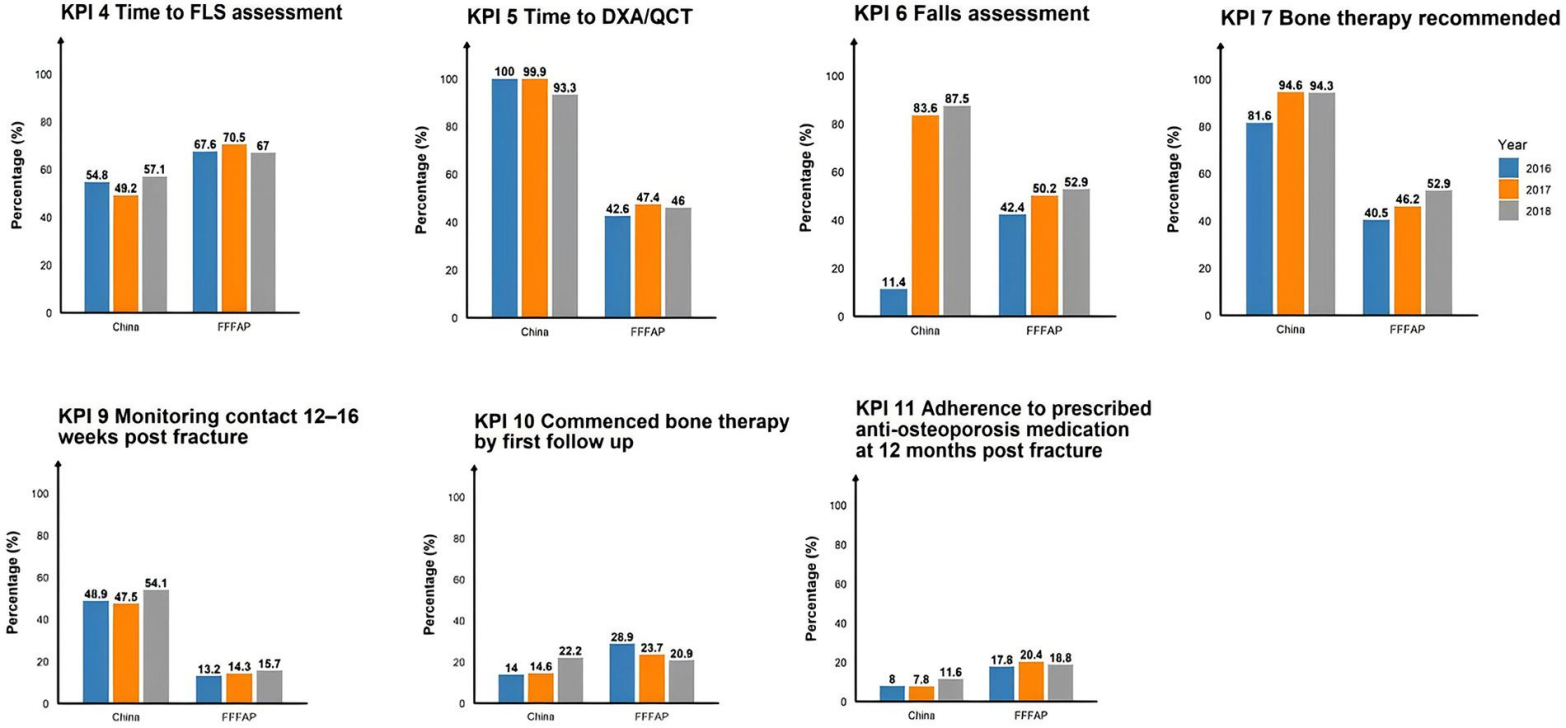
Comparative performance on selected FLS-DB KPIs between the Chinese cohort and the UK FFFAP benchmark (2016–2018).

Figure 4 presents the comparative performance of our hospital's secondary fracture prevention program against United Kingdom benchmark standards from the Fracture Liaison Service Database. Several key findings emerged from this international comparison:

The percentage of patients identified for secondary fracture prevention (KPI 4) in our hospital remained lower than the UK benchmark. For bone mineral density assessment (KPI 5), DXA-only completion rates were initially high but declined sharply by 2018, falling below UK benchmarks in the final year. When QCT was incorporated alongside DXA, assessment rates kept near-universal and consistently exceeded the UK level in all three years. Falls assessment rates (KPI 6) improved markedly over time, surpassing the UK average from 2017 onward. The proportion of patients recommended anti-osteoporosis medication (KPI 7) increased and remained above UK figures. Follow-up completion within 16 weeks post-fracture (KPI 9) consistently outperformed UK data. Bone protection treatment prescription rates at follow-up (KPI 10) showed steady improvement, achieving parity with UK standards by 2018. However, medication adherence at 12 months (KPI 11) remained notably lower than UK benchmarks.

Detailed quantitative data for all KPI comparisons are provided in Appendix Table 1.

## Discussion

This benchmark audit systematically applies—for the first time in China—the established UK Fracture Liaison Service Database Key Performance Indicators (FLS-DB KPIs) to reveal a distinct performance profile for secondary fracture prevention within the tertiary hospital system. Our findings demonstrate that while the hospital-centric orthogeriatric co-management pathway achieves excellent inpatient care metrics—particularly bone mineral density screening (97.1%) and medication initiation (94.0%)—significant gaps persist in long-term patient engagement and medication adherence. This study provides crucial evidence for adapting internationally validated FLS frameworks to China's unique healthcare delivery context, offering a roadmap for systematic quality improvement in secondary fracture prevention.

Our hospital's bone health assessment performance substantially exceeded international benchmarks due to the integration of routine quantitative computed tomography (QCT) scanning within the inpatient co-management protocol (10, 11). Notably, DXA-only completion fell to 14.1% by 2018, as QCT gradually supplanted standalone DXA. This shift reflected a pragmatic response to clinical realities: positioning an acutely injured older adult on a DXA table for the required 15 min proved logistically burdensome and uncomfortable for patients, whereas QCT offered a validated alternative for bone density assessment that gained increasing acceptance in China. The combined DXA/QCT metric therefore remained consistently high throughout the study period. This approach differs from Western FLS models where DXA scans are typically ordered during admission but completed in outpatient settings within 12–16 weeks. While UK services achieve approximately 80% DXA ordering rates (12, 13), and Australian programs report 75%–85% screening completion (14), our inpatient completion model—whether by DXA or QCT—achieved near-universal coverage, reflecting the accessibility advantages of hospital-based screening of China.

Falls risk assessment demonstrated progressive improvement, surpassing UK benchmarks from 2017 onwards through bedside multidisciplinary evaluations involving geriatricians, rehabilitation physicians, and physical therapists. This hospital-based approach mirrors successful Japanese FLS models (15, 16) but diverges from UK and Australasian systems (12, 14) where comprehensive falls assessments are deferred to community services. The effectiveness of completing assessment within the hospital reflects the concentrated expertise available in Chinese tertiary hospitals, yet also highlights the absence of robust community-based fall prevention networks to support patients post-discharge.

Anti-osteoporosis medication initiation rates reached 97.9% by 2018, exceeding UK rates (56% by 16 weeks) (12) and Australian programs (72% inpatient recommendation, 65% community prescribing at 12–16 weeks) (14). However, long-term adherence remained problematic, with only 9.5% completing 12-month follow-up, substantially below benchmarks from the UK (74%–78%) (13), Australia (60%–70%) (14), and Japan (60%) (17). This disparity between excellent initiation and poor persistence reflects fundamental structural differences: Western FLS models integrate nurse-led coordinators and community-based monitoring systems, while China's hospital-centric approach lacks systematic primary care engagement or community-level medication management support. Furthermore, median therapy duration was under two months for both calcium/vitamin D and calcitonin, confirming that treatment discontinuation occurs early after discharge. In our care pathway, post-discharge coordination consisted solely of a printed discharge summary given to patients with instructions to return for hospital-based follow-up; no formal referral was made to community health centers, and no mechanism existed for primary care providers to monitor or reinforce adherence. This gap highlighs the necessity of building robust community-based support systems (18).

Taken together, this benchmarking audit reveals both inpatient achievements and post-discharge deficits. The high inpatient screening and treatment rates observed here may not be broadly generalized, as they reflect the protocols of a well-resourced healthcare facility. However, the post-discharge attrition observed indicates a broader structural deficit: unlike integrated FLS in developed countries, China's acute hospital-driven model lacks an integration with primary healthcare facilities, leading to discontinuous rehabilitation and low adherence of anti-osteoporotic medicine. This structural limitation is compounded by patient preferences for high-level hospital care, inadequate community rehabilitation resources, and the absence of dedicated FLS coordinators who typically ensure care continuity in international models. These systemic weaknesses underscores the need for greater involvement of primary healthcare (PHC) facilities in long-term secondary prevention. Future multi-center studies would help clarify the national scope of this gap. Although our data are from 2016 to 2018, rehabilitation protocols have not changed and community-based services remain weak in China; our conclusions retain current relevance. Strengthening community rehabilitation is a priority. Sustained quality improvement will require regular audit to monitor progress and guide refinement of care pathways. Equally essential are dedicated FLS coordinators and stronger primary care integration to support long-term adherence in an aging population.

## Limitations

This study has several limitations. **First,** incomplete data capture in our hospital information system prevented comprehensive assessment of all 11 KPIs, particularly those requiring community-level outcome tracking. **Second,** as a single-center analysis, external validity may be limited, and patient outcomes managed in other healthcare facilities were not captured, potentially affecting the interpretation of the overall outcomes. **Third,** our hospital-based rehabilitation services lacked systematic post-discharge continuity, precluding collection of post-discharge mortality data and limiting assessment of long-term functional outcomes compared to community-integrated FLS models. **Fourth**, the absence of dedicated FLS coordinators, standard in international programs, may have contributed to suboptimal patient engagement and follow-up completion rates.

## Conclusion

This pioneering comparison between Chinese hospital-centric co-management and international FLS standards reveals both achievements and critical gaps in secondary fracture prevention. The observed improvements in falls assessment and medication initiation stem from progressive refinement of the orthogeriatric co-management protocol, including mandatory geriatric consultation and targeted staff education. While excellent inpatient care delivery demonstrates the potential of orthogeriatric collaboration, sustainable secondary prevention requires systematic integration of hospital and community services. Future initiatives must prioritize establishing dedicated FLS coordinator positions, developing digital health platforms for patient engagement, strengthening primary care partnerships for medication monitoring, and creating community-based rehabilitation pathways. These evidence-based adaptations are essential for scaling effective secondary fracture prevention across China's rapidly aging population while leveraging the strengths of the existing tertiary hospital infrastructure.

## Data Availability

The raw data supporting the conclusions of this article will be made available by the authors, without undue reservation.

## Appendix Table 1 Comparison of FLS-DB key performance indicator (KPI)s for all patients with an index fragility fracture.

**Table:**

KPI		China 2016 *N* = 664	China 2017 *N* = 864	China 2018 *N* = 1,161	FFFAP2016 *N* = 43,579	FFFAP2017 *N* = 52,091	FFFAP2018 *N* = 61,219
KPI 4—Time to FLS assessment The percentage of patients who were assessed by the FLS within 90 days of their fracture		54.8	49.2	57.1	67.6	70.5	67.0
KPI 5—Time to DXA The percentage of patients who had a DXA ordered or recommended and were scanned within 90 days of fracture	DXA-only	99.6	99.3	14.1	42.6	47.4	46.0
	DXA or QCT	100.0	99.9	93.3			
KPI 6—Falls assessment The percentage of patients who received a falls assessment or were referred or recommended for a falls assessment		11.4	83.6	87.5	42.4	50.2	52.9
KPI 7—Bone therapy recommended The percentage of patients who were recommended anti-osteoporosis medication		81.6	94.6	94.3	40.5	46.2	52.9
KPI 9—Monitoring contact 12–16 weeks post fracture The percentage of patients who were followed up within 16 weeks of their fracture		48.9	47.5	54.1	13.2	14.3	15.7
KPI 10—Commenced bone therapy by first follow up The percentage of patients who had commenced (or were continuing) anti-osteoporosis medication within 16 weeks of their fracture		14.0	14.6	22.2	28.9	23.7	20.9
KPI 11—Adherence to prescribed anti-osteoporosis medication at 12 months post fracture The percentage of patients who had confirmed adherence to a prescribed anti-osteoporosis medication at 12 months post fracture		8.0	7.8	11.6	17.8	20.4	18.8

KPI, key performance indicators; FLS-DB, fracture liaison service database; FFFAP, the falls and fragility fracture audit programme; DXA, dual energy x-ray absorptiometry; GP, general practitioner.
