# Supplementary material for: International benchmarking of secondary fracture prevention in older adults with hip fracture: a Chinese hospital-based KPI audit
Source: Front Health Serv. 2026 May 14;6:1799272. doi: 10.3389/frhs.2026.1799272 (PMC13216484; doi:10.3389/frhs.2026.1799272)
Supplement: Supplementary file 1 [file Table1.docx]

**STROBE Statement**

| **Section/Topic** | **Item No.** | **Checklist Item** | **Where Reported in Manuscript (Page/Line No.)** |
| --- | --- | --- | --- |
| **TITLE & ABSTRACT** | **1** | **(a) Indicate the study's design with a commonly used term in the title or abstract (b) Provide in the abstract an informative and balanced summary of what was done and what was found** | **Title, Page 1 Abstract, Page 3** |
| **INTRODUCTION** | **2** | **Explain the scientific background and rationale for the investigation being reported** | **Introduction, Page 4–5** |
|  | **3** | **State specific objectives, including any prespecified hypotheses** | **Introduction, Page 5 (study objectives stated; no prespecified hypothesis as this is a descriptive audit)** |
| **METHODS** | **4** | **Present key elements of study design early in the paper** | **Methods, Page 6** |
|  | **5** | **Describe the setting, locations, and relevant dates, including periods of recruitment, exposure, follow-up, and data collection** | **Methods, Page 7** |
|  | **6** | **Give the eligibility criteria, and the sources and methods of selection of participants.** | **Methods, Page 7** |
|  | **7** | **Clearly define all outcomes, exposures, predictors, potential confounders, and effect modifiers. Give diagnostic criteria, if applicable** | **Methods, Page 8–9**  **Table1, 9–11** |
|  | **8** | **For each variable of interest, give sources of data and details of methods of assessment (measurement). Describe comparability of assessment methods if there is more than one group** | **Methods, Page 7**  **Table1, 9–11** |
|  | **9** | **Describe any efforts to address potential sources of bias** | **Methods, Page 6 (Ethical Approval and Reporting Guidelines, i.e., anonymization, STROBE adherence)**  **Methods, Page 8–9 (predetermined exclusions of KPIs)** |
|  | **10** | **Explain how the study size was arrived at** | **Methods, Page 7** |
|  | **11** | **Explain how quantitative variables were handled in the analyses. If applicable, describe which groupings were chosen and why** | **Methods, Page 11 (No arbitrary grouping of continuous variables was performed)**  **Table 1, Page 9–11** |
|  | **12** | **(a) Describe all statistical methods, including those used to control for confounding (b) Describe any methods used to examine subgroups and interactions (c) Explain how missing data were addressed (d) If applicable, describe analytical methods taking account of sampling strategy (e) Describe any sensitivity analyses** | **Methods, Page 11  Not Applicable Complete case analysis (no imputation performed) Not Applicable Not Applicable** |
| **RESULTS** | **13** | **(a) Report numbers of individuals at each stage of the study—eg numbers potentially eligible, examined for eligibility, confirmed eligible, included in the study, completing follow-up, and analysed (b) Give reasons for non-participation at each stage (c) Consider use of a flow diagram** | **Results, Page 12–13 Reasons not systematically recorded Figure 2, Page 12** |
|  | **14** | **(a) Give characteristics of study participants (eg demographic, clinical, social) and information on exposures and potential confounders (b) Indicate number of participants with missing data for each variable of interest** | **Table 2, Page 13; Results, Page 12–13 Not reported (complete case analysis)** |
|  | **15** | **Report numbers of outcome events or summary measures** | **Results, Page 14–16**  **Figure 3, Page 14; Figure 4, Page 16** |
|  | **16** | **(a) Give unadjusted estimates and, if applicable, confounder-adjusted estimates and their precision (eg, 95% confidence interval). Make clear which confounders were adjusted for and why they were included (b) Report category boundaries when continuous variables were categorized (c) If relevant, consider translating estimates of relative risk into absolute risk for a meaningful time period** | **Not applicable (descriptive audit only)**  **Not applicable (continuous variables not categorized)**  **Not applicable (no relative risk estimates calculated)** |
|  | **17** | **Report other analyses done—eg analyses of subgroups and interactions, and sensitivity analyses** | **Not Applicable** |
| **DISCUSSION** | **18** | **Summarise key results with reference to study objectives** | **Discussion, Page 16** |
|  | **19** | **Give a cautious overall interpretation of results considering objectives, limitations, multiplicity of analyses, results from similar studies, and other relevant evidence** | **Discussion, Page 17–19** |
|  | **20** | **Discuss the generalisability (external validity) of the study results** | **Discussion, Page 19** |
| **OTHER INFORMATION** | **21** | **Give the source of funding and the role of the funders for the present study and, if applicable, for the original study on which the present article is based** | **Funding, Page 22** |
